# Association of anemia and mineral and bone disorder with health-related quality of life in Asian pre-dialysis patients

**DOI:** 10.1186/s12955-016-0477-8

**Published:** 2016-06-24

**Authors:** Hwee-Lin Wee, Benjamin Jun Jie Seng, Jia Jia Lee, Kok Joon Chong, Pallavi Tyagi, Anantharaman Vathsala, Priscilla How

**Affiliations:** Department of Pharmacy, Faculty of Science, National University of Singapore, Block S4A, 18 Science Drive 4, Singapore, 117543 Singapore; Department of Rheumatology and Immunology, Singapore General Hospital, Singapore, Singapore; Department of Medicine (Division of Nephrology), National University Hospital, Singapore, Singapore

**Keywords:** Pre-dialysis, Anemia, Mineral and bone disorder, Health-related quality of life, Pill burden

## Abstract

**Background:**

Patients with chronic kidney disease (CKD) have poor health-related quality of life (HRQoL). The association of CKD-related complications such as anemia and mineral and bone disorders (MBD) with HRQoL in pre-dialysis patients is not well-studied. As such, this study aimed to determine the association of anemia and MBD with HRQoL in pre-dialysis patients.

**Methods:**

This was a cross-sectional study involving 311 adult pre-dialysis patients with stage 3–5 CKD from an acute-care hospital in Singapore. Patients’ HRQoL were assessed using Kidney Disease Quality of Life Short Form (KDQOL-SF™) and EuroQol 5 Dimensions–3 levels (EQ5D-3L). HRQoL between patients with and without anemia or MBD were compared by separate hierarchical multiple linear regression analyses using various HRQoL scales as dependent variables, adjusted for sociodemographic, clinical and psychosocial variables.

**Results:**

After adjusting for MBD, anemia was associated with lower HRQoL scores on work status (WS), physical functioning (PF) and role physical [β (SE): −10.9 (4.18), *p* = 0.010; −3.0 (1.28), *p* = 0.018; and −4.2 (1.40), *p* = 0.003, respectively]. However, significance was lost after adjustments for sociodemographic variables. Patients with MBD had poorer HRQoL with respect to burden of kidney disease, WS, PF and general health [(β (SE): −7.9 (3.88), *p* = 0.042; −9.5 (3.99), *p* = 0.018; −3.0 (1.22) *p* = 0.014; −3.6 (1.48), *p* = 0.015, respectively]. Although these remained significant after adjusting for sociodemographic variables, significance was lost after adjusting for clinical variables, particularly pill burden. This is of clinical importance due to the high pill burden of CKD patients, especially from medications for the management of multiple comorbidities such as cardiovascular and mineral and bone diseases.

**Conclusions:**

Neither anemia nor MBD was associated with HRQoL in our pre-dialysis patients. Instead, higher total daily pill burden was associated with worse HRQoL. Medication reconciliation should therefore be routinely performed by clinicians and pharmacists to reduce total daily pill burden where possible.

## Background

Chronic kidney disease (CKD) is a serious public health problem affecting 12.8 % of Singapore’s population [1]. With the increasing prevalence of diabetes and an aging population, the number of CKD patients in Singapore is expected to rise [2].

Anemia and mineral and bone disorders (MBD) are two common complications of CKD that have been shown to increase morbidity and mortality in CKD patients [3, 4]. Anemia is caused by decreased erythropoietin synthesis or iron deficiency, and is reflected by a reduction in hemoglobin (Hgb) [5]. Mineral and bone disorder results from abnormalities in calcium (Ca), phosphorous (P), vitamin D and intact parathyroid hormone (iPTH) homeostasis and high turnover bone disease is predominant in CKD patients with secondary hyperparathyroidism [5]. Clinical presentation of MBD includes bone pain, fractures and extraskeletal calcification.

Health-related quality of life (HRQoL) has become increasingly recognized as an important outcome of medical treatment over the past two decades [6]. It is a multi-faceted measure of the impact of diseases on patients’ perceptions of their mental and physical functioning [7]. Anemia has been shown to be associated with impaired HRQoL in both pre-dialysis and dialysis patients and treatment with erythropoiesis-stimulating agents (ESAs) resulted in the improvement of HRQoL [8–10]. Suboptimal serum P and iPTH levels, as well as high pill burden from phosphate binders have also been associated with poorer HRQoL in hemodialysis (HD) patients [11–13]. In contrast, studies examining the impact of MBD on HRQoL of pre-dialysis patients are lacking. Furthermore, most studies that examined HRQoL in dialysis and pre-dialysis patients were conducted in the United States (U.S.) and Europe. Cross-cultural differences in clinical practice patterns and HRQoL may limit the generalizability of their results to an Asian country such as Singapore.

As the effect of CKD-associated complications such as anemia and MBD on HRQoL in our local CKD patients is not well-established, this study was conducted with the primary objective to determine the association of anemia and MBD with HRQoL in pre-dialysis patients in Singapore. We hypothesized that patients with anemia or MBD would have poorer HRQoL scores than patients without these complications.

## Methods

This study was approved by the National Healthcare Group Domain Specific Review Board, which is our local Institutional Review Board. Informed consent was obtained from the participants prior to the commencement of the study.

### Study design and subjects

In this cross-sectional study conducted from November 2011 to November 2013, pre-dialysis patients in the National University Hospital (NUH) outpatient renal clinic self-administered a set of survey questionnaires including the Kidney Disease and Quality of Life Short Form version 1.30 (KDQOL-SF™), EuroQol 5 Dimensions-3 Levels (EQ5D-3L) and the Medical Outcomes Family Functioning Measure (FFM). Written informed consent was obtained.

Patients aged 21 years or older with stages 3, 4 or 5 CKD, based on the Kidney Disease Quality Outcomes Initiative (KDOQI) guidelines [14] and estimated glomerular filtration rate (eGFR) calculated using the 4-variable Modification of Diet in Renal Diseases (MDRD) equation, were included in the study, as the prevalence of anemia and MBD is higher in this patient group [15–17]. Additionally, patients had to be able to read or write one of the 3 languages – English, Chinese or Malay. Patients with stage 1 or 2 CKD, on renal replacement therapy, or had impaired cognitive function were excluded from the study.

The patients’ socio-demographic information such as age, gender, race, income and education level, as well as their medical and medication histories were collected. Relevant clinical data associated with anemia and MBD such as serum Hgb, P, adjusted Ca and albumin concentrations were also collected from their electronic medical records. Adjusted Ca was calculated using serum albumin concentration if it was unavailable [18].

### Survey instruments

#### KDQOL-SF™

The KDQOL-SF™ comprises a generic and kidney disease-specific component for assessing HRQoL [19] and has demonstrated reliability and validity among dialysis patients in Singapore [20, 21]. The generic component is a 36-item health survey (SF-36) which evaluates eight aspects of HRQoL, namely physical functioning (PF), role-physical (RP), bodily pain (BP), general health (GH), vitality (VT), social functioning (SF), role-emotional (RE) and mental health (MH) [22]. Responses are transformed to a 100-point scale using the algorithm in the KDQOL-SF™ user manual [23], and by replacing the U.S. general population normative data with that of Singapore’s general population [24]. The eight scale scores are usually condensed into two normalized summary measures, namely the physical component summary (PCS) and mental component summary (MCS). However, a recently published study reported that a three-factor structure comprising of PCS, MCS and social component summary (SCS) was more appropriate for SF-36 version 2 in Singapore [25]. Another study from Singapore also reported that a three-factor structure compared to two-factor structure was more appropriate for Short-Form 12 items of the KDQOL-36 questionnaire, a shorter version of KDQOL-SF™ [26]. Hence, the KDQOL-SF™ PCS and MCS scores are inappropriate for the Singapore population and are not reported in this paper.

The disease-specific component of KDQOL-SF™ consists of 11 domains. Two dialysis-related domains were omitted as they were not relevant to our pre-dialysis patients. The remaining nine domains of disease-specific concerns were 1) symptoms, 2) effects of kidney disease, 3) burden of kidney disease, 4) work status, 5) cognitive function, 6) quality of social interaction, 7) sexual function, 8) sleep, and 9) social support. Responses were similarly transformed to a 100-point scale as described above. Higher scores indicated better HRQoL in both the generic and disease-specific components of KDQOL-SF™.

#### EQ5D-3L

EQ5D-3L is a generic HRQoL instrument which assesses the health status of individuals [27]. It has been used in several studies to measure HRQoL in pre-dialysis patients [28–30]. The instrument and its translated versions in Malay and Chinese have also been validated in Singapore [31, 32].

The EQ5D-3L comprises of a health descriptive component and a visual analogue scale (VAS) [27]. The 5-item descriptive component assesses domains of health related to mobility, self-care, usual activities, pain/discomfort and anxiety/depression. Each domain has 3 possible responses namely: no problems, some/moderate problems and extreme problems. A total of 243 possible health states can be generated from the descriptive component and the responses can be converted into a single utility score. As EQ5D-3L values for Singapore’s population are unavailable, preference weights from the U.S. population were used to derive the utility score [33]. The index score for U.S. EQ5D-3L ranges from −0.11 for worst possible health state to 1.00 for perfect health state.

The VAS is a 20-cm vertical “thermometer” which is scored from 0 to 100 points. A score of 100 represents the “best imaginable health state” while zero represents “worst imaginable health state”. VAS scores can be used as a measure of clinical outcome, utilizing the judgment of individual respondents.

#### FFM

The FFM is a 3-item instrument which evaluates the quality of interactions between respondents and their family members [34–36] in three areas of family interactions, including the level of cohesion, support/understanding and communication. Higher scores (range 0–100) indicates better family function.

#### Treatment of missing values in questionnaires

Scores for KDQOL-SF™ domains with missing values were calculated by averaging the values of completed items if values for more than half of the items in the domain were available, as recommended in the scoring manual [23]. Otherwise, the score for the domain was recorded as missing. For EQ5D-3L and FFM, missing values were left as blank [27, 37].

### Study definitions

Patients were categorized as having anemia if their Hgb concentrations were less than 10.0 g/dL or if they were receiving ESAs (epoetin alfa/beta, darbepoetin, or methoxy polyethylene glycol-epoetin beta) or oral iron supplements (ferrous gluconate or ferrous fumarate). The Hgb cutoff was based on the U.S. Food and Drug Agency’s safety alert and the Kidney Disease: Improving Global Outcomes (KDIGO) guidelines [38, 39]. They recommended initiation of ESA therapy to be individualized in pre-dialysis patients with Hgb levels below 10.0 g/dL, after taking into account patient-related factors such as response to iron therapy and the rate of Hgb fall. This was in view of higher Hgb levels being associated with increased risk of adverse events [40].

Patients were categorized as having MBD if their adjusted serum Ca and P concentrations were not within target ranges recommended by KDIGO or if they were receiving phosphate binders (Ca carbonate, Ca acetate, sevelamer hydrochloride or lanthanum carbonate at doses used for hyperphosphatemia) or vitamin D therapy (ergocalciferol, cholecalciferol, calcitriol, alfacalcidol or paricalcitol). The target range for adjusted Ca and P are 8.4–10.2 mg/dL and 2.7–4.6 mg/dL, respectively, based on our institution’s laboratory ranges [41].

The number of types of medication was defined as the number of oral, inhaled, ophthalmic, otic, topical and injectable medications each patient was on. The daily pill burden was defined as the number of oral pills taken by patients every day [11]. Medications that were taken “as needed” were not included in these computations.

### Sample size

Using α = 0.05, power = 80 % and providing for 20 % missing data, the target sample size required to detect a minimally clinically important difference (MCID) of 5 points in KDQOL-SF™ scores between patients with and without the complications was 77 patients per group. The MCID is defined as the minimal difference in scores within a domain of interest which patients view as beneficial and warrant a change in patient’s management without resulting in unnecessary costs and increased side effects [42]. It is largely accepted as a 3- to 5-point difference for KDQOL-SF™ scores [43, 44].

### Statistical analyses

Age, gender and race of patients included and excluded from the study were compared as these were the only variables collected during the preliminary screening of eligible patients. Characteristics of patients with and without anemia or MBD were also compared. Continuous and categorical variables were expressed as mean (SD) and frequencies with percentages, respectively. Continuous variables were compared using Student’s t-test or Mann Whitney-U test while categorical variables were compared using Chi-Square test or Fischer’s Exact test, where appropriate.

In univariate analyses, Student’s t-test was used to compare the HRQoL scores between patients with and without anemia and between patients with and without MBD. The association between anemia and MBD with HRQoL were evaluated in bivariate analyses. Separate multiple linear regression analyses was subsequently performed with individual HRQoL scales as dependent variable and (1) anemia as the independent variable while adjusting for other known confounders of HRQoL and (2) replacing anemia with MBD. The confounders were included using a hierarchical approach: (i) sociodemographic variables: centered age, gender, race (Chinese/Malay/Indians/Others), marital status (single/married/divorced, separated or widowed), education level (no education/primary/secondary/tertiary) and monthly income level (no income/<$2000/$2000-$3999/≥$4000); (ii) sociodemographic plus clinical variables including CKD stage (3/4/5), daily pill burden and the presence of comorbidities (yes/no). Comorbidities considered include hypertension, hyperlipidemia, diabetes, heart disease and stroke; (iii) sociodemographic and clinical plus psychosocial variables: FFM. Patients with missing data on variables used in multiple linear regression were further excluded listwise during analyses.

All statistical analyses were carried out using Stata version 12.0 (Stata Corporation, College Station, Texas).

## Results

**Fig. 1:**
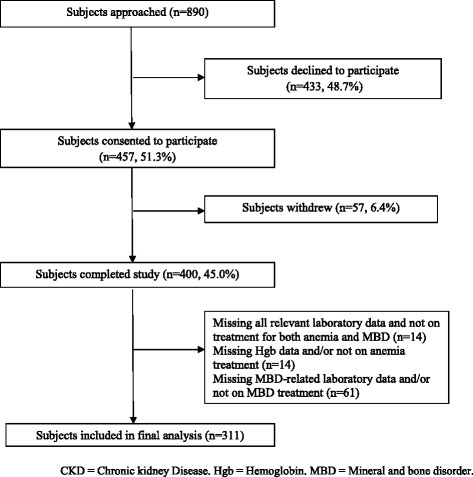
Flowchart of patient recruitment and exclusion

Of 890 subjects who were approached, 433 (48.7 %) subjects declined study participation because they felt tired or they found the surveys to be overly lengthy (Fig. 1). Another 57 withdrew midway as they felt the survey was too long. Eventually, 400 (45.0 %) subjects completed the study. Among those who completed, a further 89 subjects were later excluded as they had missing laboratory data and/or were not receiving treatment for either anemia or MBD. Hence, data from 311 subjects were eventually analysed (response rate: 68.2 % of all subjects who agreed to participate).

### Patient characteristics

Subjects who provided complete data for analyses (*n* = 311) compared to those who declined, withdrew or had incomplete data (*n* = 579) were younger [mean (SD) age, years: 62.6 (11.06) vs. 65.7 (11.42), *p* < 0.001], and more likely to be Malay (*n* = 103, 33.1 % vs. *n* = 128, 22.1 %, *p* = 0.005) (Table 1).

**Table 1:** Socio-demographic, clinical and psychosocial characteristics of patients who provided complete data for analyses and those who were excluded from the study^a^

Characteristics	Included patients (*n* = 311)	Excluded patients^a^ (*n* = 579)
Mean (SD) Age, in years	62.6 ± 11.06	65.7 ± 11.42***
Gender, N (%)		
Female	130 (41.8)	268 (46.3)
Male	181 (58.2)	311 (53.7)
Race, N (%)		
Chinese	188 (60.5)	412 (71.2)
Malay	103 (33.1)	128 (22.1)**
Indian	14 (4.5)	29 (5.0)
Others	6 (1.9)	10 (1.7)
Marital Status, N (%)		N/A
Single	28 (9.0)	
Married	220 (70.7)	
Divorced, separated or widowed	63 (20.3)	
Education level, N (%)		N/A
No education	64 (20.7)	
Primary	99 (31.9)	
Secondary	87 (28.1)	
Tertiary	60 (19.4)	
Monthly income level, N (%)		N/A
No income	198 (64.1)	
< $2000	65 (21.0)	
$2000–$3999	29 (9.4)	
$4000–$5999	10 (3.2)	
$6000–$9999	7 (2.3)	
CKD stage, N (%)		N/A
3	105 (33.8)	
4	147 (47.3)	
5	59 (19.0)	
Co-morbidities, N (%)		N/A
Hyperlipidemia	275 (88.4)	
Hypertension	278 (89.4)	
Diabetes	217 (69.8)	
Heart Disease	136 (43.7)	
Stroke	38 (12.2)	
Laboratory variables, Mean (SD)		N/A
Serum hemoglobin, g/dL	11.9 ± 1.91	
Serum phosphorous, mg/dL	4.0 ± 0.96	
Adjusted calcium, mg/dL	9.2 ± 0.48	
Medication use, Mean (SD)		N/A
Types of medication	8.4 ± 3.27	
Daily pill burden	13.0 ± 6.71	
FFM, Mean (SD)	65.5 ± 27.18	N/A

*CKD* Chronic Kidney Disease, *FFM* Family Functioning Measure, *N/A* Not available

Results are expressed as mean ± standard deviation or number (%)

Statistical significance: **p < 0.01, ***p < 0.001

^a^Excluded patients refer to those who declined participation and those who consented but subsequently withdrew from the study. Only age, gender and race were available for excluded patients as they did not provide consent for access to their medical records or did not complete the questionnaires

Patients with anemia were mostly female, had no formal education, were not earning an income, and had more advanced CKD (stage 4 or 5) (Table 2). They also had higher serum P, lower Hgb and lower adjusted Ca concentrations, were taking more types of medication and had higher daily pill burden. These differences were also observed in patients with MBD compared to those without.

**Table 2:** Significantly different characteristics between patients with and without anemia or MBD

Characteristics	All patients (*n* = 311)		All patients (*n* = 311)	
	Without anemia (*n* = 186)	With anemia (*n* = 125)	Without MBD (*n* = 166)	With MBD (*n* = 145)
Gender, N (%)				
Female	57 (30.7)	73 (58.4)***	63 (38.0)	67 (46.2)
Male	129 (69.4)	52 (41.6)	103 (62.1)	78 (53.8)
Education level, N (%)				
No education	29 (15.6)	35 (28.2)**	35 (21.2)	29 (20.0)
Primary	55 (29.6)	44 (35.5)	45 (27.3)	54 (37.2)
Secondary	60 (32.3)	27 (21.8)	51 (30.9)	36 (24.8)
Tertiary	42 (22.6)	18 (14.5)	34 (20.6)	26 (17.9)
Monthly income level, N (%)				
No income	105 (56.8)	93 (75.0)**	100 (60.6)	98 (68.1)
< $2000	42 (22.7)	23 (18.6)	33 (20.0)	32 (22.2)
$2000–$3999	24 (13.0)	5 (4.0)	20 (12.12)	9 (6.3)
$4000–$5999	8 (4.3)	2 (1.6)	8 (4.9)	2 (1.4)
$6000–$9999	6 (3.2)	1 (0.8)	4 (2.4)	3 (2.1)
CKD stage, N (%)				
3	85 (45.7)	20 (16.0)***	79 (47.6)	26 (17.9)***
4	82 (44.1)	65 (52.0)	77 (46.4)	70 (48.3)
5	19 (10.2)	40 (32.0)	10 (6.0)	49 (33.8)
Laboratory variables, Mean (SD)				
Serum hemoglobin, g/dL	12.7 ± 1.53	10.6 ± 1.66***	12.4 ± 1.71	11.2 ± 1.90***
Serum phosphorous, mg/dL	3.7 ± 0.74	4.3 ± 1.18***	3.7 ± 0.46	4.3 ± 1.24***
Adjusted calcium, mg/dL	9.2 ± 0.44	8.8 ± 0.56**	9.2 ± 0.36	8.8 ± 0.60***
Medication use, Mean (SD)				
Types of medication	7.4 ± 2.96	9.8 ± 3.23***	7.2 ± 3.00	9.7 ± 3.10***
Daily pill burden	11.1 ± 5.92	15.9 ± 6.81***	10.8 ± 5.68	15.5 ± 6.92***

*CKD* Chronic Kidney Disease

Results are expressed as mean ± standard deviation or number (%)

Statistical significance: **p < 0.01, ***p < 0.001

### Self-reported HRQoL

The self-reported HRQoL is presented in Table 3. As expected, both groups of patients generally had poorer HRQoL compared to the Singapore general population, which by default has a mean score of 50. The greatest impairment experienced by our pre-dialysis patients was on the SF-36 general health scale, with the score difference being close to 10 points (one standard deviation).

**Table 3:** Self-reported HRQoL of all patients analyzed

HRQoL scales	No. of patients	Mean (SD)	Mean^b^ (Joshi et al. 2010) [21]
Kidney disease-specific			
Symptoms	310	85.4 (17.38)	N/A
Effects of Kidney Disease	309	85.4 (19.00)	N/A
Burden of Kidney Disease	304	59.7 (33.99)	N/A
Work Status	311	49.2 (35.35)	N/A
Cognitive Function	311	85.2 (18.14)	N/A
Quality of Social Interaction	311	83.7 (18.85)	N/A
Sexual Function	113	78.1 (28.67)	N/A
Sleep	311	67.9 (21.79)	N/A
Social Support	311	80.1 (22.69)	N/A
SF-36 Scores^a^			
Physical functioning (PF)	310	45.3 (10.81)	47.47
Role-physical (RP)	309	45.6 (11.75)	44.53
Bodily pain (BP)	311	48.9 (12.37)	49.95
General health (GH)	311	40.1 (13.10)	38.83
Vitality (VT)	311	45.4 (13.06)	46.53
Social Functioning (SF)	311	50.5 (11.98)	45.22
Role-emotional (RE)	309	48.8 (10.91)	49.91
Mental health (MH)	311	52.7 (11.82)	49.13
EQ-5D utility	309	0.8 (0.24)	N/A
EQ-VAS	296	69.0 (18.70)	N/A

^a^Individual scales of the SF-36 are normalized against the Singapore general population, which has a mean score of 50

^b^Norm-based scores constructed from the raw mean scores reported by Joshi et al. [21] N/A – Not available

### Self-reported HRQoL of patients with and without anemia or MBD

In univariate analyses, patients with anemia reported significantly lower HRQoL scores on the work status item, [beta coefficient (standard error, SE): −12.7 (4.03), *p* = 0.002], SF-36 PF [beta coefficient (SE): −3.6 (1.24), *p* = 0.003] and RP [beta (SE): −4.4 (1.34), *p* = 0.001]. In bivariate analyses, after adjusting for MBD, patients with anemia persisted to have significantly lower work status [beta (SE): −10.9 (4.18), *p* = 0.010], SF-36 PF [beta (SE): −3.0 (1.28), *p* = 0.018) and RP (beta (SE): −4.2 (1.40), *p* = 0.003] scores. These scores were no longer significantly different between patients with and without anemia after adjusting for sociodemographic variables.

In univariate analyses, those with MBD reported significantly lower scores on burden of kidney disease [beta (SE): −7.9 (3.88), *p* = 0.042], work status [beta (SE): −9.5 (3.99), *p* = 0.018], SF-36 PF [beta (SE): −3.0 (1.22) *p* = 0.014] and GH [beta (SE): −3.6 (1.48), *p* = 0.015]. After adjusting for anemia, the association between MBD and burden of kidney disease [beta (SE): −8.2 (4.03), *p* = 0.043], as well as SF-36 GH [beta (SE): −3.2 (1.54), *p* = 0.038] remained statistically significant. These associations remained statistically significant after adjusting for sociodemographic variables but were no longer significant after adjusting for sociodemographic and clinical variables. In fact, statistical significance was lost simply by adjusting for pill burden. Results of these analyses are shown in Table 4.

**Table 4:** Multiple linear regression to evaluate the association of anemia or MBD with HRQoL in pre-dialysis patients

	With anemia^a^			With MBD^b^		
HRQoL scales	Univariate analyses	Bivariate analyses^c^	Multiple linear regression analyses^d^	Univariate analyses	Bivariate analyses^c^	Multiple linear regression analyses^e^
Kidney disease-specific						
Symptoms	0.8	N/A	N/A	−2.4	N/A	N/A
Effects of Kidney Disease	0.4	N/A	N/A	−3.9	N/A	N/A
Burden of Kidney Disease	−1.1	N/A	N/A	−7.9*	−8.2*	−4.5
Work Status	−12.7**	−10.9*	−3.36	−9.5*	−6.6	N/A
Cognitive Function	2.0	N/A	N/A	1.3	N/A	N/A
Quality of Social Interaction	2.7	N/A	N/A	−0.1	N/A	N/A
Sexual Function	−4.9	N/A	N/A	−5.6	N/A	N/A
Sleep	0.7	N/A	N/A	−1.8	N/A	N/A
Social Support	−0.9	N/A	N/A	−1.9	N/A	N/A
SF-36 Scale Scores						
PF_NBS	−3.7**	−3.0*	−2.2	−3.0*	−2.2	−1.7
RP_NBS	−4.4**	−4.2**	−2.7	−2.0	N/A	N/A
BP_NBS	−1.8	N/A	N/A	1.0	N/A	N/A
GH_NBS	−2.5	N/A	N/A	−3.6*	−3.2*	−2.9
VT_NBS	−2.9	N/A	N/A	−1.1	N/A	N/A
SF_NBS	−0.6	N/A	N/A	−2.5	N/A	N/A
RE_NBS	−2.1	N/A	N/A	−0.8	N/A	N/A
MH_NBS	−0.4	N/A	N/A	0.2	N/A	N/A
EQ-5D utility	−0.03	N/A	N/A	N/A	N/A	N/A
EQ-VAS	−1.7	N/A	N/A	N/A	N/A	N/A

Results are presented as beta coefficient

^a^Reference group: No anemia; ^b^Reference group: No MBD

^c^Adjusted for MBD or anemia, where appropriate

^d^Adjusted for sociodemographic variables: centered age, gender, race (Chinese/Malay/Indians/Others), marital status (single/married/divorced, separated or widowed), education level (no education/primary/secondary/tertiary) and monthly income level (no income/<$2000/$2000-$3999/≥$4000)

^e^Adjusted for sociodemographic variables plus clincal variables: CKD stage (3/4/5), the presence of co-morbidities (yes/no) and daily pill burden. Comorbidities included are hypertension, hyperlipidemia, diabetes, heart disease and stroke

*p < 0.05, **p < 0.01; N/A: Bivariate and multiple linear regression analyses were not applicable as univariate analyses were not statistically significant; NBS: norm-based score

## Discussion

In our study, after adjusting for covariates, pre-dialysis patients with anemia or MBD had similar HRQoL as those without these complications. This observation was somewhat surprising given that previous studies involving dialysis patients had suggested that these complications were associated with poorer HRQoL. The differences in unadjusted SF-36 PF and RP scores between patients with and without anemia in our study were explained by sociodemographic variables. Interestingly, the differences in unadjusted SF-36 PF and GH scores between patients with and without MBD were explained by total daily pill burden alone. Our patients with MBD had significantly higher mean total daily pill burden (15.5 vs. 10.8, Table 3). In post-hoc univariate analyses, total daily pill burden was associated with all SF-36 scales except RE and MH. After adjusting for sociodemographic and clinical variables (excluding MBD), the association between total daily pill burden and SF-36 scales remained statistically significant for PF, GH, VT and SF.

Another possible explanation for the small effect of MBD on HRQoL is that although complications associated with MBD such as vascular calcification and bone pain are expected to diminish patients’ physical and mental functioning, the prevalence of these complications is higher in patients with more advanced CKD or those undergoing dialysis [45, 46]. Such complications may have been subclinical in our sample with predominantly stages 3 and 4 CKD (83.8 %). Additionally, patients with MBD may not experience symptoms until much later when more serious complications such as fractures, cardiovascular morbidity and mortality arise.

This study has several important strengths. First, based on PubMed search using keywords “mineral bone disorder” AND “quality of life” or “mineral bone disease” AND “quality of life” on 06 Aug 2014, this is the first study that evaluated the impact of MBD on HRQoL of pre-dialysis CKD patients. Current literature on the effects of MBD on HRQoL is limited to the dialysis population where derangements in serum P and i-PTH (both low and elevated levels), as well as low 25-hydroxyvitamin D [25(OH)D] levels have been associated with poor HRQoL [12, 13, 47]. Since MBD develops as early as at moderate CKD, knowledge of its effects on HRQoL of patients with stage 3-4 CKD would be useful. Furthermore, early management of MBD has the potential to reduce cardiovascular complications [48]. In our study, at least, it appears that while MBD impairs HRQoL, the choice of MBD treatment with regards to daily pill burden will have a direct and perhaps more prominent impact on HRQoL compared to MBD itself. An association between high pill burden, mostly from phosphate binders, and lower HRQoL was also reported in a study involving 233 dialysis patients in the US [11]. Nonetheless, the reporting of total daily pill burden is not common among HRQoL studies. Our finding is important as it suggests that total daily pill burden is often overlooked as an important factor affecting patient’s HRQoL. In two studies, vitamin D supplementation with ergocalciferol or cholecalciferol did not improve HRQoL in dialysis patients [49, 50]. Perhaps the HRQoL gained from optimal MBD management had been offset by the HRQoL impairment associated with increased pill burden. Thus, more research is needed in this area to better inform the choice of MBD treatments and their impact on HRQoL.

Second, this is one of very few studies that evaluated the impact of anemia on HRQoL in pre-dialysis patients [51]. In a study among 69 pre-dialysis CKD patients, patients with anemia reported poorer Short-Form 12 MCS compared to patients without anemia [52]. However, in our study, anemia had no significant impact on HRQoL in pre-dialysis patients. This may suggest cultural differences in the impact of anemia on HRQoL. Alternatively, the differences may be due to different characteristics of patients in the two studies. While it is encouraging that pre-dialysis patients with anemia are able to carry out as much physical activities and feel as energetic as those without anemia, we need to be aware of its potential impact on adherence to anemia treatment. As patients may not directly experience improvement in HRQoL arising from the treatment of anemia, adherence may be poor.

We recognized that this study is not without its limitations. First, although our study included a wide spectrum of relevant variables in our regression analyses, other variables associated with impaired physical and mental HRQoL in pre-dialysis patients such as poor nutritional status, depression and inflammation were not captured [53, 54]. As the assessment of nutritional status and depression would require administration of additional questionnaires, this could impose excessive cognitive burden on our patients, which would in turn affect the quality of data. Furthermore, inflammatory markers such as C-reactive protein are not routinely measured in our centre. Nonetheless, Farag et al reported that regardless of adjustment for inflammatory markers, patients with anemia had worse Short-Form 12 MCS than patients without anemia [52]. Hence, the effects of inflammation on HRQoL may have been “masked” by the effects of anemia on HRQoL. It is unclear if the effects of malnutrition and depression on HRQoL would similarly be “masked” by the effects of anemia. Future studies are needed to better elucidate the relationship of these modifiable factors that can help improve HRQoL of CKD patients. Second, the cross-sectional design of the study precludes the establishment of a cause and effect relationship between anemia, MBD and HRQoL. Nonetheless, our results serve as a basis for future prospective longitudinal studies to explore any causal relationships between anemia and MBD with HRQoL. Third, selection bias is likely to exist as patients were not randomly selected. Furthermore, those who completed the study were younger and had a higher proportion of Malays compared to those who declined, withdrew or had incomplete data. Hence, this may limit the generalizability of our findings. Last but not least, due to the large percentage of missing data for iPTH (50.0 %) and 25(OH)D levels (68.8 %), these two parameters were not included as part of our study’s definition for MBD. While this could have led to potential misclassification of patients, the problem was minimised as patients with suboptimal iPTH and 25(OH)D levels were likely to have been on treatment for MBD, which was encompassed in our definition of MBD. Fourth, the EQ-5D utility and EQ-VAS scores were not significantly different between patients with and without anemia or MBD. This may reflect a lack of sensitivity of the EQ-5D questionnaire for detecting small differences. As such, for the purpose of comparing health preferences between patients with and without anemia or MBD, the sensitivity of an alternative health preference questionnaire such as the Short-Form 6 Dimension or the Health Utilities Index will need to be evaluated for this population.

## Conclusions

Our study has shown that anemia is not associated with HRQoL in pre-dialysis patients. While MBD as a whole is also not significantly associated with HRQoL, total daily pill burden in pre-dialysis patients with MBD is associated with HRQoL. This is of clinical importance due to the recognized high pill burden of CKD patients, especially from medications prescribed for the management of hyperphosphatemia, secondary hyperparathyroidism and comorbidities such as cardiovascular diseases. Clinicians and pharmacists should review patients’ medication lists regularly and perform medication reconciliation routinely to reduce their total daily pill burden, where possible. Future research is also needed to examine effective therapeutic options for MBD that does not compromise HRQoL due to excessive pill burden.

## Abbreviations

Beta, Beta coefficient; BP, Bodily pain; Ca, Calcium; CKD, Chronic kidney disease; EQ5D-3L, EuroQol 5 Dimensions–3 levels; ESA, erythropoiesis-stimulating agent; FFM, Family functioning measure; GH, General health; Hgb, Hemoglobin; HRQoL, Health-related quality of life; iPTH, intact-parathyroid hormone; KDIGO, Kidney Disease: Improving Global Outcomes; KDOQI, Kidney Disease Quality Outcomes Initiative; KDQOL-SF™, Kidney Disease Quality of Life Short Form; MBD, Mineral and bone disorder; MCID, Minimally clinically important difference; MCS, Mental component summary; MDRD, Modification of Diet in Renal Diseases; MH, Mental health; NUH, National University Hospital; P, Phosphorus; PCS, Physical component summary; PF, Physical functioning; RE, Role-emotional; RP, Role physical; SCS, Cocial component summary; SD, standard deviation; SE, standard error; SF, Social functioning; U.S., United States; VT, Vitality; WS, Work status
